# *In Silico* Analysis of Putative Paralytic Shellfish Poisoning Toxins Export Proteins in Cyanobacteria

**DOI:** 10.1371/journal.pone.0055664

**Published:** 2013-02-15

**Authors:** Katia Soto-Liebe, Xaviera A. López-Cortés, Juan José Fuentes-Valdes, Karina Stucken, Fernando Gonzalez-Nilo, Mónica Vásquez

**Affiliations:** 1 Pontificia Universidad Católica de Chile, Santiago, Chile; 2 Nanobiotechnology Division at University of Talca, Fraunhofer Chile Research Foundation - Center for Systems Biotechnology, Talca, Chile; 3 Institute of Molecular Evolution Heinrich-Heine, Universität Düsseldorf, Düsseldorf, Germany; 4 Universidad Andres Bello, Center for Bioinformatics and Integrative Biology, Santiago, Chile; Tel Aviv University, Israel

## Abstract

Paralytic shellfish poisoning toxins (PSTs) are a family of more than 30 natural alkaloids synthesized by dinoflagellates and cyanobacteria whose toxicity in animals is mediated by voltage-gated Na^+^ channel blocking. The export of PST analogues may be through SxtF and SxtM, two putative MATE (multidrug and toxic compound extrusion) family transporters encoded in PSTs biosynthetic gene cluster (*sxt*). *sxtM* is present in every *sxt* cluster analyzed; however, *sxtF* is only present in the *Cylindrospermopsis-Raphidiopsis* clade. These transporters are energetically coupled with an electrochemical gradient of proton (H^+^) or sodium (Na^+^) ions across membranes. Because the functional role of PSTs remains unknown and methods for genetic manipulation in PST-producing organisms have not yet been developed, protein structure analyses will allow us to understand their function. By analyzing the *sxt* cluster of eight PST-producing cyanobacteria, we found no correlation between the presence of *sxtF* or *sxtM* and a specific PSTs profile. Phylogenetic analyses of SxtF/M showed a high conservation of SxtF in the *Cylindrospermopsis-Raphidiopsis* clade, suggesting conserved substrate affinity. Two domains involved in Na^+^ and drug recognition from NorM proteins (MATE family) of *Vibrio parahaemolyticus* and *V. cholerae* are present in SxtF/M. The Na^+^ recognition domain was conserved in both SxtF/M, indicating that Na^+^ can maintain the role as a cation anti-transporter. Consensus motifs for toxin binding differed between SxtF and SxtM implying differential substrate binding. Through protein modeling and docking analysis, we found that there is no marked affinity between the recognition domain and a specific PST analogue. This agrees with our previous results of PST export in *R. brookii* D9, where we observed that the response to Na^+^ incubation was similar to different analogues. These results reassert the hypothesis regarding the involvement of Na^+^ in toxin export, as well as the motifs L^398^XGLQD^403^ (SxtM) and L^390^VGLRD^395^ (SxtF) in toxin recognition.

## Introduction

Cyanobacteria are a biochemically and morphologically diverse clade of photosynthetic bacteria, with major environmental and economic roles. They are main primary producers in marine and freshwater ecosystems, key biocatalysts in the N_2_ cycle [1] and some are capable of producing toxins. Toxin production can present waterborne health hazards for humans and animals. This has become of particular interest lately, due to the proliferation and dominance of harmful blooms of cyanobacteria; a result of the increase of eutrophication and climate change among others [2]. In addition, toxic strains of *Anabaena*, *Anabaenopsis*, *Microcystis* and *Nodularia* are salt tolerant, and increasing halotolerant HABs have been observed more frequently [2].

Major cyanobacterial toxins include microcystins, cylindrospermopsins, nodularin, anatoxins and saxitoxins (STX) [3]. STX and its analogues (more than 30) have been detected in filter-feeding bivalve mollusks. In humans, these toxins cause paralytic shellfish poisoning (PSP). PSP-toxins (PSTs) produce symptoms that vary from a slight tingling sensation or numbness around the lips to a fatal respiratory paralysis. The long-established molecular target of PSTs is the voltage-gated sodium channel in nerve and muscle cells, to which PSTs bind with analogue-dependent affinity, blocking Na^+^ channels in nanomolar concentrations [4]. STX has also been shown to target voltage-gated potassium [5] and calcium [6] ion channels.

**Figure 1:**
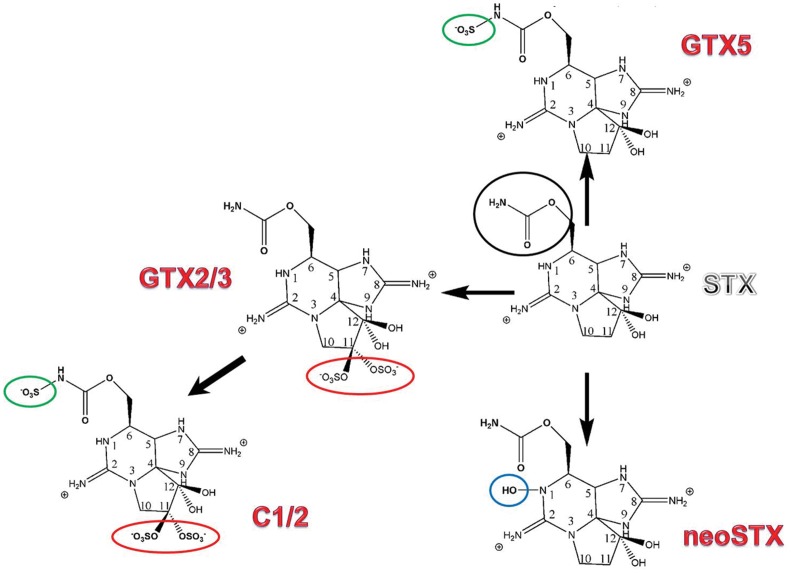
Final saxitoxin (STX) analogues synthesized by cyanobacteria (as described in [21]). Variations of the structure of saxitoxin are circled: carbamoyl group (black), sulfate group (red), sulfonate group (green) and hydroxyl group (blue).

STX is a trialkyltetrahydropurine molecule (Figure 1), with two pKa’s of 8.22 and 11.28 in aqueous solution, which belong to the 7,8,9 and 1,2,3 guanidinium groups, respectively. At physiological pH, the 1,2,3-guanidine carry a positive charge, whereas the 7,8,9-guanidine group is partially deprotonated. STX and its analogs can be structurally classified into several classes such as non-sulfated (STX, neoSTX), mono-sulfated (gonyautoxins-GTX1–6), di-sulfated (C1–4), and decarbamoylated, each with varying levels of toxicity. Of the decarbamoyl variants of these analogs, there are decarbamoyl-saxitoxins (dcSTX, dcneoSTX) and decarbamoyl-gonyautoxins (dcGTXs 1–4).

In the past decades, the PSTs-Na^+^ channel binding process has been well studied and modeled [7], [8], indicating that only one PST molecule is bound per Na^+^ channel. Sodium (Na^+^) is one of the most predominant soluble ions in saline soils and waters. It is also an important requirement for cyanobacterial growth [9], [10] and nitrogen fixation [11].

The use of a Na^+^ gradient turns out to be highly efficient in cyanobacteria when taking into account that in *Anabaena torulosa* (brackish water strain) and *Anabaena* L-31 (freshwater strain), intracellular Na^+^ concentration remains lower than in the culture medium: 10 and 30 times lower (ΔψNa^+^ = ^+^79.7–^+^90.5 mV) in *Anabaena torulosa* and 11 and 18 times lower (ΔψNa^+^ = ^+^66.5–^+^75.7 mV) in *Anabaena* L-31 under extracellular sodium concentration ranges between 1 and 60 mM [11].

**Figure 2:**
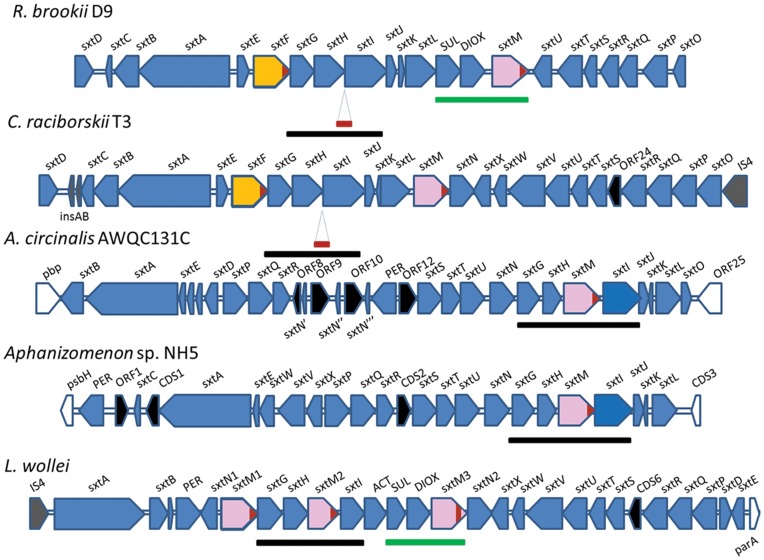
Comparative analysis of five *sxt* clusters in cyanobacteria . ORFs *sxtF* and *sxtM* are shown in yellow and pink, respectively. Homologous segments are indicated in black (*sxtG/H/M/I*) and green (*sxtSUL/DIOX/M*). Putative scare sequence present in intergenic region *sxtH-sxtI* and also identified in the end of *sxtF*, *sxtM* sequences, is shown in red. ORFs with unknown functions are shown in black, ORFs flanking the clusters in white and the transposases element in grey.

To date, five PSTs biosynthetic gene clusters (*sxt*) have been described in cyanobacteria *Cylindrospermopsis raciborskii* T3, *Anabaena circinalis* AWQC131C, *Aphanizomenon* sp. NH5, *Lyngbya wollei* and *Raphidiopsis brookii* D9 [12], [13], [14], [15]. Cluster *sxt* size ranges between 25.7 kb (*R. brookii*) and 36 kb (*L. wollei*) (Figure 2). Among *sxt* genes, two have been related to toxin export: *sxtF* and *sxtM*, which encode a MATE (multidrug and toxic compound extrusion) transporter. This protein family, represented by NorM in the bacterium *Vibrio parahaemolyticus,* confers resistance to multiple cationic toxic agents. These proteins export norfloxacin (NOR) and other cationic toxic compounds by means of an electrochemical gradient of Na^+^ ions, acting as a Na^+^/drug antiporter [16]. In clusters involved in cylindrospermopsin synthesis (*cyr*) *cyrK* is present, a gene that also encodes a MATE protein belonging to the NorM family [15]. The conserved regions G^184^KFGXP^189^ and L^381^RGYKD^386^ present in NorM of *Vibrio parahaemolyticus*, *V. cholerae* and other bacteria have been characterized as recognition motifs for Na^+^ and drugs respectively. The first domain was located between transmembrane (TM) domains V-VI and the second one between X-XI TM domains [16].

Both the binding of STX with the Na^+^ channel and the interaction of NOR with the NorM protein, have demonstrated the key role of negatively charged residues of transporter proteins in STX blocking [7] and NOR resistance [16], respectively.

Although the effect of PSTs have been well documented in regards to human health, their role in phytoplankton ecology is less clear. However, these studies are subject to speculation, where PSTs could be synthesized as a chemical defense mechanism and/or for ion transport facilitation and regulatory interactions [17]. Recently, an interaction between STX and the copper transporter Ctr1p has been proposed, suggesting that STX inhibited copper uptake [18]. In previous work, we established a relationship between the extracellular levels of Na^+^ and the export of PSTs in the cyanobacterium *Raphidiopsis brookii* D9 [19]. We proposed that PSTs in cyanobacteria could act as a protective mechanism to ensure homeostasis against extreme salt variation in the environment. This phenomena has been reported in a combination of evaporated and groundwater intrusions [20].

PST-producing cyanobacteria synthesized one analogue in major proportion, which was named “final analogue” [21]. Analogs differ in side group moieties (Figure 1), but guanidinium groups are maintained in all analogues.

In this study we developed a different approach to understand why there is more than one copy of putative transporter proteins in the sequenced *sxt* clusters, considering previous data on PST profiles in cyanobacteria and the export of toxins in *R. brookii* D9. Finally, our aim was to correlate the data obtained from mutagenesis studies of the NorM protein of *V. cholerae* with molecular docking modeling analysis of SxtF/M proteins with specific PSTs analogues.

## Materials and Methods

### Isolation and Culture Conditions

*Raphidiopsis brookii* D9 was obtained by sub-cloning from the culture SPC338 (provided by Maria Teresa de Paiva, Sao Paulo, Brazil), as originally isolated by Pedro Zagatto from a branch of the Billings water reservoir in Taquacetuba, Sao Paulo, Brazil. *Cylindrospermopsis raciborskii* MVCC14 (provided by S. Bonilla, Instituto de Investigaciones Biológicas Clemente Estable, Montevideo, Uruguay) was isolated from a shallow lake in Uruguay. *C. raciborskii* ITEP A3 [22] and *C. raciborskii* PMC0.01 [22] were obtained from the Paris Museum Collection (provided by Cécile Bernard). The isolation sites and toxin profiles are described in Table 1. Cells were grown in batch cultures in MLA medium at pH 8.4 [23] at 25°C, under continuous cool-white fluorescence lighting at a photon flux density of 75 µE s^−1^ m^−2^.

**Table 1:** PSTs profiles, presence of *sxtM/F* transporter genes and source of cyanobacterial strains.

	PSTs profiles							Transporters		
Strain	STX	GTX2/3	dcSTX	dcGTX2/3	neoSTX	C1/2	LW1–6	*sxtF*	*sxtM*	References
CR T3^(1)^	+				++			+	+	[^21^] ^a; ^ [^12^] ^b^
CR PMC00.01^(1)^	+				++			+	+	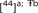
CR ITEP A3^(1)^	+				++			+	+	Bernard Per. 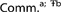
CR MVCC14(2)	+	+ +	+	+				+	+	Fuentes-Valdés, 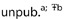
Raph D9^(1)^	+	++	+	+				+	+	[^21^] ^a; ^ [^15^] ^b^
LW(3)			+	+			++	?	+	[^45^] ^a; ^ [^46^] ^a; ^ [^14^] ^b^
AC AWQC131C(4)	+	+	+	+		++			+	[^47^] ^a; ^ [^13^] ^b^
Apha NH5(3)	+				+				+	[^48^] ^a^; [^13^] ^b^

a Toxin profile.

b Gene sequences.

? Classification is not clear.

+ Gene or toxin is present.

++ PST analogue is synthesized in higher proportion (final analogue).

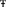
This work.

CR: *C. raciborskii*; Raph: *Raphidiopsis*; Apha: *Aphanizomenon*; AC: *A. circinalis*; LW: *L. wollei.*

Sources: ^(1)^Brazil,

(2) Uruguay,

(3) USA,

(4) Australia.

### Genomic DNA Isolation, Amplification, Sequencing and Phylogeny

DNA was extracted with the CTAB method described by Wilson [24]. For PCR amplification and the sequencing of *sxtF/M* from *C. raciborskii* ITEP A3, *C. raciborskii* PMC00.01, *C. raciborskii* MVCC14, the primers described in Table S1 were used. The PCR reaction contained 50–100 ng of genomic DNA. Reagents for each amplification were: 0.25 U Taq DNA polymerase (Invitrogen®, California, USA); 3 µL10X PCR buffer (Invitrogen®); 2.5 mM MgCl (Invitrogen®, California, USA); 0.4 mM primers; and 0.93 mM of each deoxynucleoside triphosphate (Promega®, Madison, Wi-USA). Thermal cycling was performed in an Eppendorf Mastercycler (Westbury, NY-USA), under the following conditions: initial DNA denaturation at 92°C for 2 min; 30 cycles at 94°C for 1 min, 56°C for 1 min, 72°C for 2 min and a final elongation at 72°C for 7 min.

PCR primers were used for the sequencing of both DNA strands (Macrogen, Korea). All sequences were manually checked using BLASTX in addition to the National Center for Biotechnology Information (NIH, Bethesda, MD). Phylogenetic and molecular evolutionary analyses were conducted using MEGA version 5 [25]. The model was chosen based on JTT because, according to options given by MEGA5, BIC scores (Bayesian Information Criterion) were lowest in consideration to the given substitution pattern (File S2). When using different substitution models the tendency remained the same. ProTest (version 2.4) supported the substitution model in this work (data not shown) [26].

To predict protein structure, we used the programs PSIPRED (http://bioinf.cs.ucl.ac.uk/psipred/) and Predict Protein (http://www.predictprotein.org/).

### GenBank Accession Numbers

The published sequences were obtained from the National Center for Biotechnology Information (NCBI) database (http://www.ncbi.nlm.nih.gov/) under the accession numbers: ABI75096 (SxtF, *C. raciborskii* T3), ABI75103 (SxtM, *C. raciborskii* T3), ACG58379 (SxtM, *A. circinalis* AWQC131C), ACG63815 (SxtM, *Aphanizomenon* sp. NH-5), ACG63829.1 (SxtM1, *Lyngbya wollei*), ACG63832.1 (SxtM2, *L. wollei*), ACZ26231.1 (SxtM3, *L. wollei*), ZP_06941036 (NorM, *Vibrio cholerae* RC385), ZP_06305235 (SxtF, *R. brookii* D9), ZP_06305227 (SxtM, *R. brookii* D9), ADA69242.1 (MatE efflux transporter, *Nostoc* sp. ‘*Peltigera membranacea* cyanobiont’), ACZ26226.1 (SxtPER, *L. wollei*), ACZ26223.1 (SxtPER, *Aphanizomenon* sp. NH-5), ABI75130.1 (SxtPER, *A. circinalis* AWQC131C). The SxtF/M protein described in this study has been deposited in the GenBank database under the accession numbers JX105885 (SxtF *C. raciborskii* ITEP A3), JX105886 (SxtF *C. raciborskii* PMC00.01), JX105887 (SxtF *C. raciborskii* MVCC14), JX105888 (SxtM *C. raciborskii* ITEP A3), JX105889 (SxtM *C. raciborskii* PMC00.01), JX105890 (SxtM *C. raciborskii* MVCC14).

### Modeling

#### Homology modeling

The reference X-ray structure used to build the five homology models was the MATE transporter NorM from *Vibrio cholerae* [27] (ID code: 3MKT; Resolution: 3,65 Å; R-Value: 0,312) which was obtained from the protein data bank (Research Collaboratory for Structural Bioinformatics, RCSB, http://www.rcsb.org/pdb).

The homology models were built for the proteins SxtM_T3, SxtF_T3, SxtM_131C, SxtM_D9 and SxtF_D9. Sequences were aligned using Clustal W [28]. After using this alignment tool and the software MODELLER version 9v6 [29], homology models were built for all the sets of proteins. The new models were subjected to cycles of energy minimization of 500 steps, and to a further molecular dynamic of 100 ps in order to relax the conformation of the lateral chains and avoid conformation tension generated during the construction of the model. All calculations were performed using the NAMD [30] and force field charm [31].

#### Docking simulations

AutoDockVina [32] was used to explore the pores of all the studied proteins (SxtM_T3, SxtF_T3, SxtM_131C, SxtM_D9 and SxtF_D9). In order to study the interaction of the different toxins throughout the pore of each transporter, a technique that separates the pore into 5 separate grids maps was used, where each grid has a 10 Å superimposition with each other along the Z axis. This system was developed so that each transporter binding site could be explored thoroughly.

For the construction of toxins STX, GTX2, GTX3, neoSTX, C1 and C2, density functional theory (DFT) methods [33] were used that considered B3LYP [34], [35] and the 3–21 g* basis set, in order to obtain the optimized molecules. These calculations were made using gaussian03 [36]. MAESTRO graphical interface and OPLS [37] force field were used to assign partial charges for the set of proteins and toxins. Autodock Tools (ADT) was used to prepare the proteins and the ligands. All water molecules were removed and the atomic solvation parameters and fragmental volumes were assigned to the protein using the AutoDockVina program. The grid maps were calculated using Autogrid module and were centered on the Z-axis of the pore. The volume chosen for the grid maps was 54×54×54 points with a grid-point spacing of 0.375 Å (20×20×20 Å). Autotors was used to define the rotatable bonds in the toxins. The Lamarckian Genetic Algorithm was used for all docking calculations. Visual inspections of the results were performed using the MGL Tools package [38].

## Results

### Phylogenetic and Sequence Analysis

**Figure 3:**
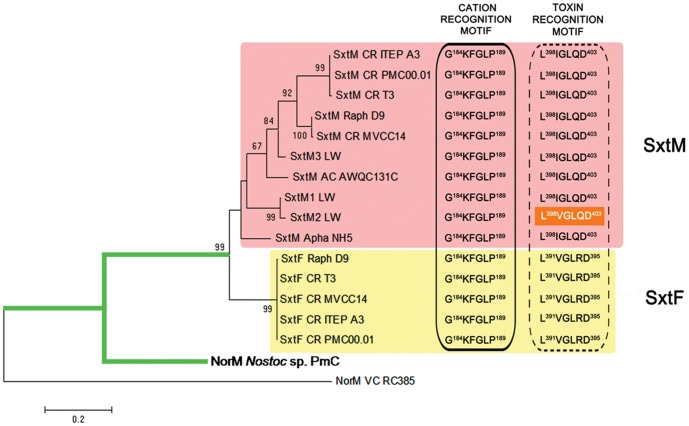
Maximum Likelihood phylogenetic tree for SxtF/SxtM and its homologues in *Nostoc* sp. and *Vibrio cholerae*. The dendrogram was inferred with a JTT amino acid substitution model, uniform rate and a bootstrap of 1000 replications (only values over 60% are shown) (MEGA version 5). The subdivisions of the tree correspond to the two different types of transporters, whose amino acid sequence in the two specific recognition motifs (Cation and Toxin), are given in the right part of the figure. The cyanobacterial branch is marked in green. SxtM recognition motifs are shown in a pink rectangle and SxtF motifs in a yellow rectangle. The toxin recognition motif of SxtM2 from *L. wollei* contains a non- expected amino acid sequence (orange rectangle), corresponding to an amino acid mixture: in position 399 there is a V (Valine) similar to SxtF toxin motif, and in position 402 there is a Q (Glutamine), similar to SxtM. CR: *C. raciborskii*; Raph: *Raphidiopsis*; Apha: *Aphanizomenon*; AC: *A. circinalis*; LW: *L. wollei* ¸ PmC: *Peltigera membranacea* cyanobiont; VC: *Vibrio cholerae*.

We identified and sequenced the putative toxin transporter genes *sxtM* and *sxtF*, of three *C. raciborskii* strains (PMC00.01, ITEP A3 and MVCC14), differing in toxin profiles (Table 1, File S1). Maximum likelihood phylogenetic inference of SxtF and SxtM amino acid sequence shows a common ancestor for both transporters in all PST-producing cyanobacteria, as well as two well supported branches: one for the SxtMs and another for the SxtFs sequences (bootstrap 99%) (Figure 3). SxtF topology lacked phylogenetic resolution (identities >99), however SxtM topology grouped the strains that produce STX-neoSTX (CR ITEP A3, CR T3, CR PMC00.01) and those that produce STX-GTX2/3 (RB D9, CR MVCC14) (Table 1).

It is important to note that both *sxtF* and *sxtM* were not identified in the non-PSTs producing cyanobacterial strain, *Raphidiopsis* sp. ITEP005 (confirmed by PCR analysis, data not shown). In addition, the sequence with highest similarity to SxtF and SxtM, found by comparison against the cyanobacterial genome database, corresponds to NorM of *Nostoc* sp., a protein present in a different gene cluster related to secondary metabolite synthesis.

By means of multiple alignments (CLUSTAL W) of SxtF and SxtM amino acid sequences from all PSTs-producing cyanobacteria and *V. cholerae* (VC), we identified that the motifs characterized as Na^+^ (G^184^KFGXP^189^) and drug (L^381^XGXXD^386^) recognition sites were conserved in SxtF and SxtM (Figure 3). The SxtM2 (L^398^ **V**GL**Q**D^403^) motif from *Lyngbya wollei*, the only cyanobacterium that does not produce STX, but the decarbamoylated form and several unique analogues (Table 1), shows a mixed sequence between the SxtM (L^398^IGL**Q**D^403^) and SxtF (L^390^ **V**GLRD^395^) motifs. Topology prediction of SxtF/M using the online servers PSIPRED and Predict Protein corroborated the periplasmic (TM V-TM VI) localization of the Na^+^ recognition and the cytoplasmic loop (TM X-TM XI) of the drug recognition domains (data not shown). The amino acid identity between NorM of VC with SxtF and SxtM of *R. brookii* D9 is 30% and 28%, respectively.

In all PST-producing cyanobacteria, where only *sxtM* was identified (but not *sxtF*) (*L. wollei, A. circinalis* AWQC131C, *Aphanizomenon* sp. NH5) in the *sxt* cluster (Figure 2, Table 1), a gene encoding for a putative transporter protein, *sxtPER*, is present. This gene is not present in another region of the *R. brookii* D9 genome. Analysis of the SxtPER sequence of three cyanobacterial strains revealed that 10 transmembrane domains are located between amino acids 318–405, whose N- and C-terminal are located at the cytoplasm (topology according to MEMSAT3 Prediction, http://bioinf.cs.ucl.ac.uk/psipred/) (Figure S1). The identified SxtPER homologues belong to the membrane protein family Pfam00892 (DUF6). These proteins were included in the cluster of the orthologous group (COG) 0697 as permeases of the drug/metabolite transporter (DMT) superfamily [39]. According to the transporter classification database (www.tcdb.org), SxtPER is classified as part of the 2.A.7 DMT superfamily, apparently fitting within the 2.A.7.3 10 TMS Drug/Metabolite Exporter (DME) Family. Considering the existence of experimental data in NorM proteins, but not in SxtPER homologues, only the SxtF/M proteins were considered for further analysis.

### Homology Modeling

Homology models were developed for the putative transporters SxtF and SxtM of *C. raciborskii* T3, *A. circinalis* AWQC131C and *R. brookii* D9 (SxtF_T3, SxtM_T3, SxtM_AWQC131C, SxtF_D9 and SxtM_D9). These proteins share between 27% and 30% identity with NorM of *Vibrio cholerae* [27], a protein with a characterized crystal structure. In order to explore the pore and binding sites of these proteins, docking simulations were performed for the systems described in Table 2. The pore of the transporter was studied by using an overlapping of 10 Å between the 5 grids of 20×20×20 Å along the Z-axis.

**Table 2:** Binding energies of the PST analogues and putative binding sites in SxtF and SxtM.

	PSTs analogues and binding energies (kcal/mol)*					
Systems	GTX2	GTX3	STX	neoSTX	C1	C2
SxtF_Raph D9	−8,3	−9,6	−8,6	NP	NP	NP
SxtM_ Raph D9	−6,9	−6,9	−8,2	NP	NP	NP
SxtF_CR T3	NP	NP	−7,5	−7,1	NP	NP
SxtM_CR T3	NP	NP	−6,8	−6,8	NP	NP
SxtM_AC AWQC131C	−6,9	−7,1	−	NP	−7,1	−7,1

* Interactions obtained from docking calculations to Grid 2.

Raph: *Raphidiopsis brookii*; CR: *C. raciborskii*; AC: *A. circinalis*.

NP: Analogue is not present.

(−): System was not analyzed.

**Figure 4:**
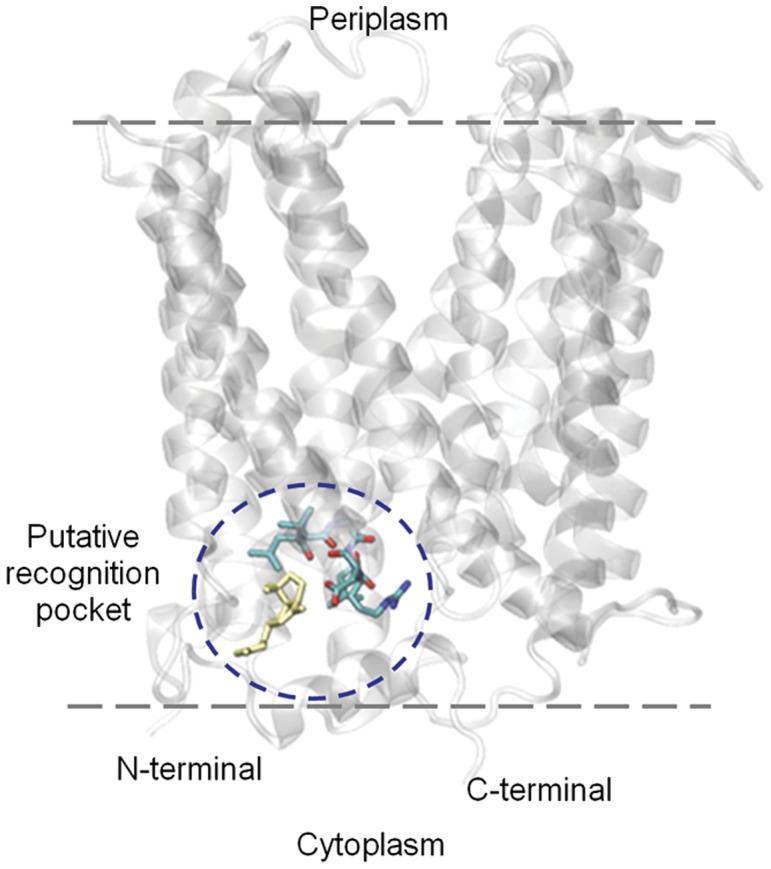
Predicted protein structure of SxtF of *R. brookii* D9, lateral view. The model was built based on the X-ray structure of the MATE transporter NorM of *Vibrio cholerae* (ID code: 3MKT; Resolution: 3,65 Å; R-Value: 0,312). The location in which we obtained the most favorable binding energies (domain ^390^VGLRD^395^ in licorice representation) is shown circled in blue. STX corresponds to the yellow colored molecule.

The most favorable binding energy was obtained for grid 2, which contains the predicted binding domains L^398^XGLQD^403^ (SxtM) or L^390^VGLRD^395^ (SxtF) (Figure 4). The energies obtained for these pockets (binding domains) are not significantly different between the analogues synthesized for each strain (Table 2), because the values fall into the error of the method [32] (see materials and methods). When comparing amongst strains, the most favorable energy was observed in the SxtF systems of *R. brookii* D9 (Table 2). We analyzed the interaction of these domains with Na^+^ and it apparently does not affect the toxin-binding domain (Figure S2).

**Figure 5:**
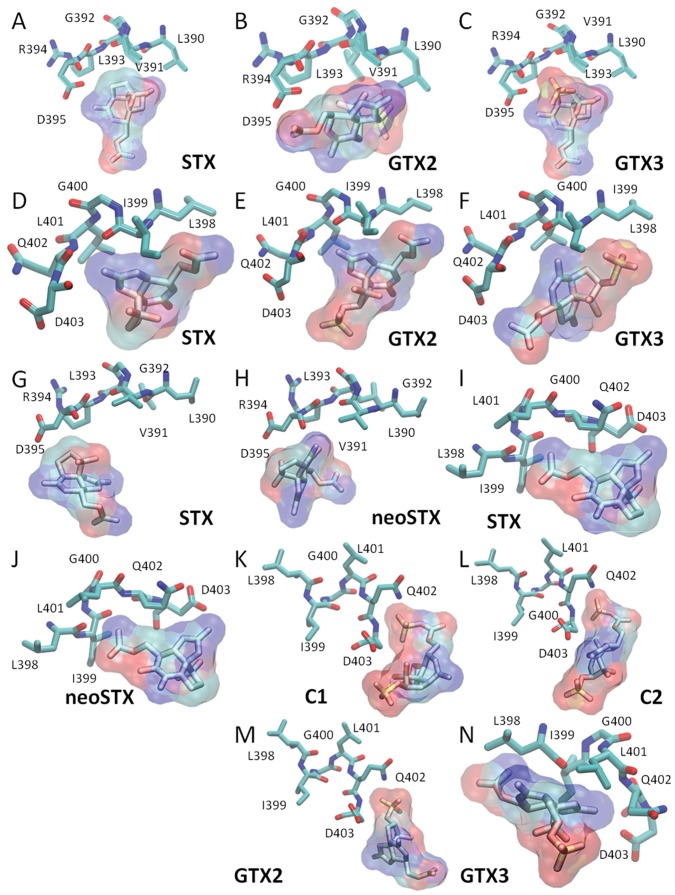
Representation view of PST-recognition sites in SxtF/M. The PST recognition region in licorice representation corresponds to: SxtF *R. brookii* D9 (A, B and C) for the conserved region LVGLRD; SxtM *R. brookii* D9 (D, E and F) for the conserved region LIGLQD; SxtF *C. raciborskii* T3 (G and H), for the conserved region LVGLRD; SxtM *C. raciborskii* T3 (I and J) for the conserved region LIGLQD; SxtM *A. circinalis* AWQC131C (K, L, M and N) for the conserved region LIGLQD. PSTs are depicted in the surfaces representations. The name of the each PST is indicated in the bottom section of the model. The licorice representation shows: carbon atoms in cyan, oxygen in red, nitrogen in blue and sulfur in yellow. Hydrogen atoms were omitted.

The specific interactions in docking models are presented in Figure 5. Overall, we observed that SxtF and SxtM systems in strains D9 and T3 are similar in terms of both backbone folding and side chain geometry in the toxin-binding site.

In the SxtF_D9 system, D^395^ interacts directly with 1,2,3 guanidinium group of STX (Figure 5 A) and GTX3 (Figure 5 C). In the SxtM_D9 system L^401^ interacts with 7,8,9 guanidinium goups of STX (Figure 5 D), GTX2 (Figure 5 E) and GTX3 (Figure 5 F). In the SxtF/M_T3 systems, we observed the interaction between D^395^ (SxtF) with 1,2,3 guanidinium group of STX (Figure 5 G) and D^403^ (SxtM) with 7,8,9 guanidinium group of STX (Figure 5 I) and neoSTX (Figure 5 J).

Finally, in the SxtM_131C system, direct interactions were between: Q^402^ with sulfocarbamoyl group of analogues C1 (Figure 5 K) and C2 (Figure 5 L); Q^402^ with sulfate group (C11) of GTX2; D^403^ with guanidinium group in C2 (Figure 5 L), GTX2 (Figure 5 M) and GTX3 (Figure 5 N).

## Discussion

To date, transformation methods applied for genetic manipulation in PST-producing cyanobacteria have yet to be developed. The functional classification of proteins encoded by *sxt* genes has been explored by sequence homology [12], [13], [14], [15] and by correlating the presence of *sxt* genes with toxin profiles in cyanobacteria [21]. The *sxt* cluster in cyanobacteria has a core of 15 genes, which has been conserved, in both sequence and synteny, among 5 clusters described to date. One of the genes present in all clusters is *sxtM* [40] (Fuentes-Valdés et al., unpublished data) (Table1, Figure 2). In previous work, we proposed that PSTs are exported in the cyanobacterium *R. brookii* D9, where SxtF/M could be involved in toxin export [19]. However, unlike *sxtM*, the *sxtF* gene is only present in the *Cylindrospermopsis*-*Raphidiopsis* clade.

With the aim of exploring the relationship between the presence of the SxtF and SxtM transporters and the export of PSTs analogues, we compared the presence of both predicted transporter genes and toxin profiles in eight PST-producing cyanobacteria. Our data shows no correlation between both aspects, preventing the prediction of specific analogue-transporter relationships. This might be explained by the presence of SxtF/M in all *Cylindrospermopsis* strains and in *R. brookii* D9, which differ in their toxin profiles. Furthermore, only SxtM is present in *A. circinalis*, *Aphanizomenon* sp. and *L. wollei*, which also posses different toxin profiles amongst each other (Table 1). In these strains we identified a second protein, SxtPER, which could act as an alternative transporter of PSTs, compensating for the lack of SxtF.

All PSTs-producing cyanobacteria, except *L. wollei*, synthesize only one analogue in a major proportion, which we previously named the “final analogue” [21] (Figure 1). In the *sxt* cluster of *R. brookii* D9, which contains the minimum set of genes required to synthesize STX and GTX2/3, the last two epimers are the final analogues. Furthermore, *sxtF* and *sxtM* are transcribed [15], thereby raising the question: why does *R. brookii* D9 keep and express more than one transporter? On the other hand, the fact that *L. wollei* synthesizes the higher number of analogues in greater proportions (LW1, LW2/3 -epimers- and LW5, which constitute the 88% of the total), and contains three copies of *sxtM* and one copy of *sxtPER* [14], suggests that there is apparently a relationship between the number of final analogues and transporter proteins.

The high conservation of SxtF (100% amino acid identity) may be ascribed to purifying selection and a recent horizontal gene transfer (HGT) event, as previously described for the cyr cluster [41]. Although the *sxt* cluster is extraordinarily conserved in cyanobacteria [42], the differences between the divergences of the SxtF and SxtM clades are notorious. Cyanobacterial phylogeny based on the 16S rRNA gene suggests that the presence of a PSTs phenotype was either gained in the toxic strains through independent HGTs or was lost from the non-toxic strains [40]; the same event has been observed in other toxic phenotypes [43]. The phylogeny of SxtMs does not completely agree with the cyanobacterial phylogenies based on the 16S rRNA gene [40] because the SxtM copies that belong to a more distant cyanobacterium, *L. wollei*, are distributed between the other sequences (Figure 3). Similar topologies were obtained for other *sxt* genes, where *L. wollei* sequences group together with the clade formed by *Cylindrospermopsis*-*Raphidiopsis* strains (data not shown). Evolutionary markers such as the 16S rRNA gene and the internal transcribed spacer ITS1 showed that the *Cylindrospermopsis-Raphidiopsis* clade from America are closely related, with over 99% of nucleotide identity [22]. The presence of two well-defined branches of STX-neoSTX (CR ITEP A3, CR T3, CR PMC00.01) and STX-GTX2/3 (RB D9, CR MVCC14) producers (Figure 3; Table 1) implies that the evolution of SxtM was dependent on the toxin profiles in this clade. However, posterior analyses are necessary to be able to contrast the evolution of other sequences belonging to the *sxt* cluster and their evolutionary markers.

Murray et al., [42] showed a different topology for the SxtF/M phylogenies based on the ML method and a CpREV+ I+G+F substitution model. While analyzing amino acid residues 1–280, they observed two branches considering that SxtM of *Aphanizomenon* sp. NH5 and SxtM1-SxtM2 of *L. wollei* were classified as a putative SxtF. Although these sequences are the most distant in the SxtM clade (Figure 3), we considered the analysis of the previously described active motif of NorM proteins in *V. cholerae* (VC), and assigned it to the SxtF/M classification.

When we analyzed domains that are putatively involved in toxin recognition, we were able to distinguish between SxtF and SxtM domains (Figures 3; File S1), with the exception of SxtM2 of *L. wollei*. This may be related to the synthesis of STX derivatives named *L. wollei* toxins 1–6 (Lw toxin 1–6), which are only present in *L. wollei*. These observations suggest that these domains would maintain the drug-toxin recognition function of SxtF/M, and the differences could indicate transporter-toxin selectivity. The domain described in VC as the one responsible for Na^+^ recognition is conserved in all SxtF/M sequences, but is less conserved in the NorM protein of *Nostoc* sp. *‘Peltigera membranacea* cyanobiont’, the sequence closer to the SxtF/M clade. This is evidence that positive selection has acted upon the G^184^KFGXP^189^ motif to possibly maintain the role of Na^+^ in transporter function. In previous studies we have analyzed the effect of Na^+^ on PSTs levels in *R. brookii* D9, concluding that Na^+^ plays an important role in toxin export [19], which is in concordance with the motif analysis.

Comparative sequence analyses have shown that several recombination events have shaped the *sxt* gene clusters, especially in the *sxtF/M* gene family [42]. Based on comparative sequence analysis, Murray et al. [42] have suggested that a common ancestor of Nostocales possessed both *sxtF* and *sxtM* orthologs but while they were kept in *R. brookii*, in a common ancestor of *A. circinalis* and *Aphanizomenon* sp. NH-5, these genes were subjected to recombination events that generated a novel copy of the gene that shared the largest fraction of its sequence for either *sxtM* (*A. circinalis*) or *sxtF* (*Aphanizomenon* sp. NH-5). In both cases this *sxtF/M* ortholog is located between the *sxtH* and *sxtI* genes.

Interestingly, our analysis shows that even though *R. brookii* D9 possesses both *sxtF* and *sxtM* orthologs, there is a 95 bp sequence between the *sxtH* and *sxtI* genes with high identity with the C-terminal region of the *sxtF/M* genes. This finding suggests that *sxtF/M* gene was present in this location at some point and that was subsequently lost. Whether there were up to three copies of the *sxtF/M* genes in a common ancestor of the Nostocales clade or that the scar between *sxtH* and *sxtI* is a remnant of an intermediate step of *sxF/M* recombination events, will only be clarified with the generation of novel genomic sequences from other representatives of the Nostocales clade.

Despite these recombination events, the putative PST-recognition site has been conserved in SxtF and SxtM and our modeling and docking analysis results reassert the hypothesis of the involvement of L^398^XGLQD^403^ (SxtM) and L^390^VGLRD^395^ (SxtF) domains in toxin recognition. The fact that the predicted binding energies are similar for all analogues and for each analyzed cyanobacterium, could indicate that SxtF/M does not have selectivity for a final analogue. This is in agreement with our observation related to the similar toxin export behavior of STX-GTX2/3 in *R. brookii* D9 (data not shown).

Regarding the binding energies between toxins and mammalian sodium channel, values obtained by Tikhonov and Zhorov [7] are very low, which explain channel blocking due to permanent binding between selectivity-filter region and toxin. In our study, interaction energies are higher, which allows temporal binding of the toxin with its recognition site and posterior export. In the binding of STX with the Na^+^ channel, glutamic and aspartic residues play a key role in channel blocking [7]. In this study we also observed interaction between aspartic residues and guanidinium groups in some PST analogues.

As aforementioned, the *sxt* cluster has undergone intra- and interspecific recombination, and has been subject to duplication, which results in differences in PST profiles amongst cyanobacteria. This is probably the reason why transporter proteins remain without particular selectivity for any analogue. On the other hand, although the role of PSTs in the environment is unknown, the interaction between PSTs and sodium channels is highly sensitive to modifications in STX structure [8]. For example, the predicted affinity between C1 and GTX3 with SxtM of *A. circinalis* AWQC131C, is identical (Table 2), but in terms of toxicity in mice, GTX3 is three orders of magnitude more toxic than C1 [8]. Therefore, there would be selective pressure to maintain the conserved toxin recognition domains in the transporters in order to recognize the variety of final analogues synthesized in different cyanobacteria. Probably, differences in analogue structures are related to their target in the environment rather than the recognition by transporter proteins SxtF and SxtM.

### Conclusions

This study suggests that the previously identified motif involved in drug recognition in the NorM protein of *Vibrio cholerae*, and conserved in SxtF/M (cyanobacteria), could actually participate in the recognition of PSTs. We propose that the role of Na^+^ in PST export described for the NorM protein of VC is maintained in the SxtF/M transporters. The binding energies obtained for all the analyzed complexes indicate that it is not possible to discriminate protein-ligand specificity through these *in silico* studies, and that STX and the final analogues maybe exported by both transporters (SxtF/M). Posterior studies based on mutagenic models of PST-producing cyanobacteria or heterologous expression of SxtF/M, are necessary to confirm the conclusion shown in this study.

The same *in silico* (modeling-docking) approximation could be employed to discover drugs capable of blocking SxtF/M, which would allow performing a broader amount of assays in cyanobacteria permitting a better understanding of toxin role under different culture conditions.

Our results also suggest that further studies are required to evaluate toxin activity outside of the cells, where they hypothetically should be acting.

## Supporting Information

Figure S1 Predicted protein structure of SxtPER of *A. circinalis* AWQC131C, obtained from MEMSAT3.
(TIF)

Figure S2 Representation view of the PST-recognition site in SxtF of *R. brookii* D9, interacting with a sodium atom (NA).
(TIF)

Table S1 PCR primers used for amplification and sequencing. The primer position is based on the *sxtF/M* sequence of *R. brookii* D9.
(DOC)

File S1 Multiple alignment of the amino acid sequence of SxtF/M in *R. brookii* D9 (Raph) and its homologs in the *C. raciborskii* (CR) strains ITEP A3, PMC00.01, MVCC14; *L. wollei* (LW); *A. circinalis* AWQC131C (AC), *Aphanizomenon* sp. NH5 (Apha); *Nostoc* sp. *Peltigera membranacea* cyanobiont (PmC) and *Vibrio cholerae* (VC), using CLUSTAL W. *, identical residues, :>60% homologous residues. The conserved region G^184^KFGXP^189^ is marked with gray and L^381^XGXXD^386^ in black. The SxtF sequences are in bold.
(DOC)

File S2 Best amino acid substitution model identified by Akaike Information Criterion (AIC).
(PDF)
