# Correction: *In Silico* Analysis of Putative Paralytic Shellfish Poisoning Toxins Export Proteins in Cyanobacteria

**DOI:** 10.1371/annotation/98980683-2c5f-4c7f-b2ab-345d558bf9b7

**Published:** 2013-09-10

**Authors:** Katia Soto-Liebe, Xaviera A. López-Cortés, Juan José Fuentes-Valdes, Karina Stucken, Fernando Gonzalez-Nilo, Mónica Vásquez

A funding organization, a grant from that organization and an additional grant from FONDECYT were incorrectly omitted from the Funding Statement.

The Funding Statement should read: "KSL is a Comisión Nacional de Investigación Científica y Tecnológica (CONICYT) PhD fellow. This work was financed and supported by Fondo Nacional de Desarrollo Científico y Tecnológico (FONDECYT) Grant 1050433,1080075, FONDEF Grant MR10I1008 and Postdoctoral FONDECYT Grant 3130681." 

