# Supplementary figures and images for: In Silico Analysis of Putative Paralytic Shellfish Poisoning Toxins Export Proteins in Cyanobacteria
Source: PLoS One. 2013 Feb 15;8(2):e55664. doi: 10.1371/journal.pone.0055664 (PMC3574068; doi:10.1371/journal.pone.0055664)

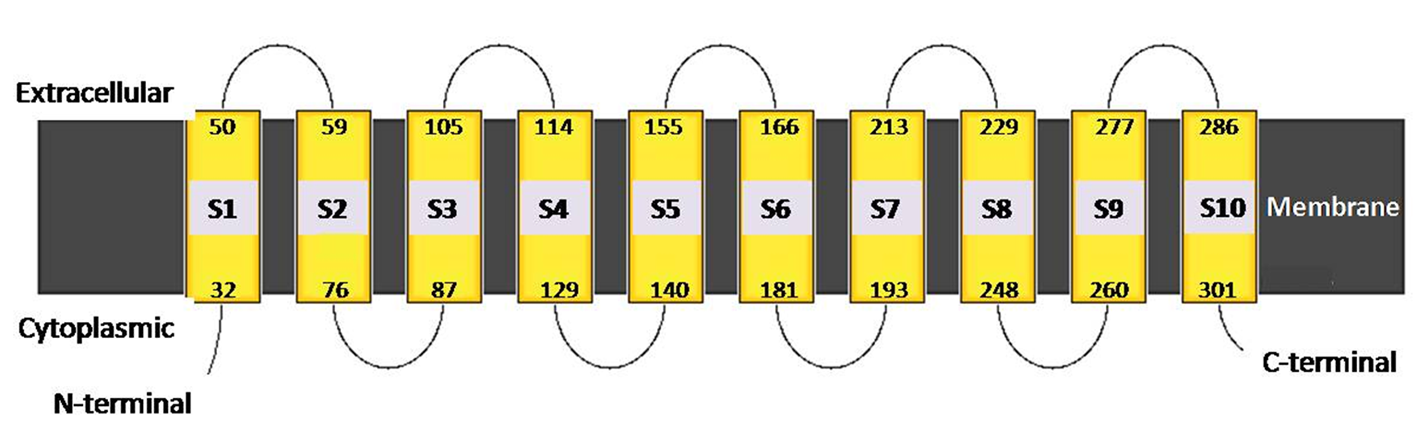

Supplement: Figure S1 — Predicted protein structure of SxtPER of A. circinalis AWQC131C, obtained from MEMSAT3. (TIF) [file pone.0055664.s001.tif]

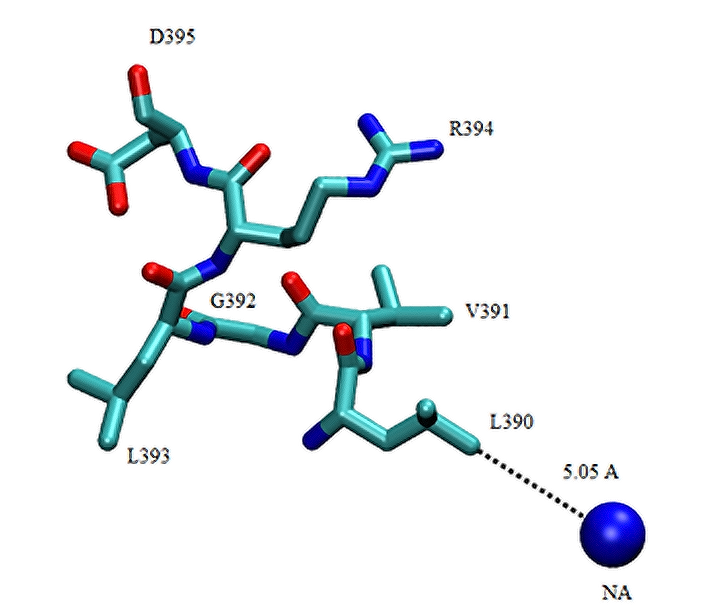

Supplement: Figure S2 — Representation view of the PST-recognition site in SxtF of R. brookii D9, interacting with a sodium atom (NA). (TIF) [file pone.0055664.s002.tif]
