# Supplementary material for: In Silico Analysis of Putative Paralytic Shellfish Poisoning Toxins Export Proteins in Cyanobacteria
Source: PLoS One. 2013 Feb 15;8(2):e55664. doi: 10.1371/journal.pone.0055664 (PMC3574068; doi:10.1371/journal.pone.0055664)
Supplement: Table S1 — PCR primers used for amplification and sequencing. The primer position is based on the sxtF/M sequence of R. brookii D9. (DOC) [file pone.0055664.s003.doc]

Table S1. PCR primers used for amplification and sequencing. The primer position is based on the *sxtF/M* sequence of *R. brookii* D9.

| **Primer *** | **Position (nt)** | **Sequence 5´- 3´** |
| --- | --- | --- |
| sxtF_D9cpF | 1-20 | ATGGAAACAACCTCAAAAAA |
| sxtF_D9cpR | 1,393-1,413 | TCAGCCTAGAGTAGTAACAG |
| sxtM_D9/20-1413F | 20-40 | GATTAGCAGAAGTAACATCA |
| sxtM_D9/20-1413R | 1,393-1,413 | CTCTTCACCATCATCACTAT |

* All primers were designed for use in this study.
