# Supplementary material for: In Silico Analysis of Putative Paralytic Shellfish Poisoning Toxins Export Proteins in Cyanobacteria
Source: PLoS One. 2013 Feb 15;8(2):e55664. doi: 10.1371/journal.pone.0055664 (PMC3574068; doi:10.1371/journal.pone.0055664)
Supplement: File S1 — Multiple alignment of the amino acid sequence of SxtF/M in R. brookii D9 (Raph) and its homologs in the C. raciborskii (CR) strains ITEP A3, PMC00.01, MVCC14; L. wollei (LW); A. circinalis AWQC131C (AC), Aphanizomenon sp. NH5 (Apha); Nostoc sp. Peltigera membranacea cyanobiont (PmC) and Vibrio cholerae (VC), using CLUSTAL W. *, identical residues, :>60% homologous residues. The conserved region G184KFGXP189 is marked with gray and L381XGXXD386 in black. The SxtF sequences are in bold. (DOC) [file pone.0055664.s004.doc]

**File S1.** Multiple alignment of the amino acid sequence of SxtF/M in *R. brookii* D9 (Raph) and its homologs in the *C. raciborskii* (CR) strains ITEP A3, PMC00.01, MVCC14; *L. wollei* (LW); *A. circinalis* AWQC131C (AC), *Aphanizomenon* sp. NH5 (Apha); *Nostoc* sp. *Peltigera membranacea* cyanobiont (PmC) and *Vibrio cholerae* (VC), using CLUSTAL W. *, identical residues, :>60% homologous residues. The conserved region G^184^KFGXP^189^ is marked with gray and L^381^XGXXD^386^ in black. The SxtF sequences are in bold.

SxtM_CR_ITEP_A3 -------LAEVTSTGYKSELRSEARVSLQLAIPLVLVEICGTSINVVDVVMMGLLGTQVL 53

SxtM_CR_T3 MTNTERGLAEITSTGYKSELRSEARVSLQLAIPLVLVEICGTSINVVDVVMMGLLGTQVL 60

SxtM_CR_PMC0001 -------LAEVTSTGYKSELRSEARVSLQLAIPLVLVEICGTSINVVDVVMMGLLGTQVL 53

SxtM_Raph_D9 MINTQNGLAEVTSKGYKSELRSEARVSLQLAIPLVVVETCETSINTVDVVMMGLLGTQVL 60

SxtM_CR_MVCC14 -------LAEVTSKGYKSELRSEARVSLQLAIPLVVVETCETSINTVDVVMMGLLGTQVL 53

SxtM3_LW MTNTQSGLAEITSTGYKSELISEARVSLQLAIPLVVVEICETSINTVDVVMMGLLGTQVL 60

SxtM2_LW MTNTQRGLAEITSTGYKSELISEARASLQLAIPLVISQMCETGINTVNVVMMGLLGTQVL 60

SxtM1_LW MTNTQRGLAEITSTGYKSELISEARASLQLAIPLVISQMCETGINTVNVVMMGLLGTQVL 60

SxtM_AC_AWQC131C MTNPQIRLAEITSKGYKSELISEARVSLQLAIPLVVVEICETSINTVDVVMMGLLGTQVL 60

SxtM_Apha_NH5 MTNPQIRLAEITSKGYKSAIISEARVSLQLAIPLVISQMCETGIYTVNAVMMGLLGTQVL 60

**SxtF_CR_T3 --------METTSKKFKSDLILEARASLKLGIPLVISQMCETGIYTANAVMMGLLGTQVL 52**

**SxtF_CR_MVCC14 --------METTSKKFKSDLILEARASLKLGIPLVISQMCETGIYTANAVMMGLLGTQVL 52**

**SxtF_CR_PMC0001 --------METTSKKFKSDLILEARASLKLGIPLVISQMCETGIYTANAVMMGLLGTQVL 52**

**SxtF_CR_ITEP_A3 --------METTSKKFKSDLILEARASLKLGIPLVISQMCETGIYTANAVMMGLLGTQVL 52**

**SxtF_Raph_D9 --------METTSKKFKSDLILEARASLKLGIPLVISQMCETGIYTANAVMMGLLGTQVL 52**

NorM_Nostoc_sp_PmC -------MEQITSTEFKSDFLTEARVSLGLAVPLAVAQLAEFTIPVINSVMMGLLGTQNL 53

NorM_VC_RC385 --------MENSVHRYK----KEASNLIKLATPVLIASVAQTGMGFVDTIMAGGVSAIDM 48

: : :* ** : *. *: : . . : : :* * :.: :

SxtM_CR_ITEP_A3 AAGALGAIAFLSVSNTCYNMLLSGVAKASEAFGANKIDQVSRIASGQIWLALTLSLPAML 113

SxtM_CR_T3 AAGALGAIAFLSVSNTCYNMLLSGVAKASEAFGANKIDQVSRIASGQIWLALTLSLPAML 120

SxtM_CR_PMC0001 AAGALGAIAFLSVSNTCYNMLLSGVAKASEAFGANKIDQVSRIASGQIWLALTLSLPAML 113

SxtM_Raph_D9 AGGALGVIAFLTLLFTCSSTLLAGGAQAAEAFGANNIDRLCRIASGNLWLATALSLPAMF 120

SxtM_CR_MVCC14 AGGALGVIAFLTLLFTCSSTLLAGGAQAAEAFGANNIDRLCRIASGNLWLATALSLPAMF 113

SxtM3_LW AGGALGAIAFLTLFFTCSSMLLAGGAQAAEAFGANNIDRVSRIASGQIWLAVALSLPAML 120

SxtM2_LW AGGALGAIAFLTLLFTCYGILSAGGAIAAEAFGANNIDQLRRIASGQIWLVVALSLPAML 120

SxtM1_LW AGGALGAIAFLTLLFTCYGILSAGGAIAAEAFGANQIDRLRRIASGQIWLVVALSLPAML 120

SxtM_AC_AWQC131C AGGALGAITFLALFFTCSGMFLVGGAQAAEAFGANKIDQVSRIASGQIWLAVALSLPAML 120

SxtM_Apha_NH5 AGGALGSLAFLNFLFTCYGITSAGGAQAAEAFGANKIDQVSRIASGQIWLAVALSLPAML 120

**SxtF_CR_T3 AAGALGALAFLTLLFACHGILSVGGSLAAEAFGANKIDEVSRIASGQIWLAVTLSLPAML 112**

**SxtF_CR_MVCC14 AAGALGALAFLTLLFACHGILSVGGSLAAEAFGANKIDEVSRIASGQIWLAVTLSLPAML 112**

**SxtF_CR_PMC0001 AAGALGALAFLTLLFACHGILSVGGSLAAEAFGANKIDEVSRIASGQIWLAVTLSLPAML 112**

**SxtF_CR_ITEP_A3 AAGALGALAFLTLLFACHGILSVGGSLAAEAFGANKIDEVSRIASGQIWLAVTLSLPAML 112**

**SxtF_Raph_D9 AAGALGALAFLTLLFACHGILSVGGSLAAEAFGANKIDEVSRIASGQIWLAVTLSLPAML 112**

NorM_Nostoc_sp_PmC AAGALGVVIFLTLASICIGILRAAGALAAEAFGANNLDRVSRINCQGLWLAVALSLPAML 113

NorM_VC_RC385 AAVSIAASIWLPSILFGVGLLMALVPVVAQLNGAGRQHKIPFEVHQGLILALLVSIPIIA 108

*. ::. :* . . .:: **.. ..: : *. :*:* :

SxtM_CR_ITEP_A3 LLWYMDTILVLFGQVESNTLLAKTYLHSIVWGFPAAVGILILRGIASAVNVPQLVTVTML 173

SxtM_CR_T3 LLWYMDTILVLFGQVESNTLIAKTYLHSIVWGFPAAVGILILRGIASAVNVPQLVTVTML 180

SxtM_CR_PMC0001 LLWYMDTILVLFGQVESNTLLAKTYLHSIVWGFPAAVGILILRGIASAENVPQLVTVTML 173

SxtM_Raph_D9 MLWHGDTILLLFGQQQSNVLLAKTYLHSIVWGFPAALGIKILRGIASAVNVPQLVTVTML 180

SxtM_CR_MVCC14 MLWHGDTILLLFGQQQSNVLLAKTYLHSIVWGFPAALGIKILRGIASAVNVPQLVTVTML 173

SxtM3_LW LLWHGDTILLLFGQEESNVLLAKTYLHSIVWGFPAAVGIKILRGIASAVNVPQLITVTML 180

SxtM2_LW LLWHTDTILLLFGQEESNVLLTKIYLQTIVWGFPAALGVMILRGIASAVNVPRLITVTML 180

SxtM1_LW LLWHTDTILLLFGQEESNVLLTKIYLQTIVWGFPAALGVMILRGIASAVNVPRLITVTML 180

SxtM_AC_AWQC131C LLWHTDTILLLLGQEESNVLVTKTYLHSIVWGFPAALGIKILRGIASAVNLTRLITVTMV 180

SxtM_Apha_NH5 LLWHGDTILLLLGQQESTVLLAKIYLQTIVWGFPAALGLLILRGIGSAVNVPQLMTVIML 180

**SxtF_CR_T3 LLWHGDTILLLFGQEESNVLLTKTYLHSILWGFPAALSILTLRGIASALNVPRLITITML 172**

**SxtF_CR_MVCC14 LLWHGDTILLLFGQEESNVLLTKTYLHSILWGFPAALSILTLRGIASALNVPRLITITML 172**

**SxtF_CR_PMC0001 LLWHGDTILLLFGQEESNVLLTKTYLHSILWGFPAALSILTLRGIASALNVPRLITITML 172**

**SxtF_CR_ITEP_A3 LLWHGDTILLLFGQEESNVLLTKTYLHSILWGFPAALSILTLRGIASALNVPRLITITML 172**

**SxtF_Raph_D9 LLWHGDTILLLFGQEESNVLLTKTYLHSILWGFPAALSILTLRGIASALNVPRLITITML 172**

NorM_Nostoc_sp_PmC LLWNCDSILPLLGQEESNILLTKSYLHVVVWGLPAMLGFLYLKQIAAAINFPQFGMVIIV 173

NorM_VC_RC385 VLFQTQFIIRFMDVEEAMATKTVGYMHAVIFAVPAYLLFQALRSFTDGMSLTKPAMVIGF 168

:*: : *: ::. :: : *:: :::..** : . *: : . ...: : .

SxtM_CR_ITEP_A3 VGLVLNAPANYVLMFGKFGLPELGLAGIGWASTLVFWISFLVGVVLLIFSPKVRDYKLFR 233

SxtM_CR_T3 VGLVLNAPANYVLMFGKFGLPELGLAGIGWASTLVFWISFLVGVVLLIFSPKVRDYKLFR 240

SxtM_CR_PMC0001 VGLVLNAPANYVLMFGKFGLPELGLAGIGWASTLVFWISFLVGVVLLIFSPKVRDYKLFR 233

SxtM_Raph_D9 VGLVLNAPANYVLMFGKFGLPELGLAGIGWASTLVFWISFLVGVVLLIFSPKVRDYQLFR 240

SxtM_CR_MVCC14 VGPVLNAPANYVLMFGKFGLPELGLAGIGWASTLVFWISFLVGVVLLIFSPKVRDYQLFR 233

SxtM3_LW VGLVLNAPANYVLMFGKFGLPQLGLAGIGWASTLVFWVSFIVGVILLIFSPKVRDYQLFR 240

SxtM2_LW AGLVLNAPADYVLMFGKFGLPQLGLAGIAWATALVFWLSFIVGVILLIFYPKVRDYKLFC 240

SxtM1_LW AGLVLNAPADYVLMFGKFGLPQLGLAGIAWATALVFWLSFIVGVILLIFYPKVRDYKLFC 240

SxtM_AC_AWQC131C LGLLLNAPANYVLMFGKFGLPQLGLAGIGWASTLVFWVSFIVGVILLIFSPKVRDYQLFR 240

SxtM_Apha_NH5 AGLILNAAADYVLMFGKFGLPQLGLVGIGWATVLVFWVSFIAGVILLIFSPKVRDYQLFR 240

**SxtF_CR_T3 TQLILNTAADYVLIFGKFGLPQLGLAGIGWATALGFWVSFTLGLILLIFSLKVRDYKLFR 232**

**SxtF_CR_MVCC14 TQLILNTAADYVLIFGKFGLPQLGLAGIGWATALGFWVSFTLGLILLIFSLKVRDYKLFR 232**

**SxtF_CR_PMC0001 TQLILNTAADYVLIFGKFGLPQLGLAGIGWATALGFWVSFTLGLILLIFSLKVRDYKLFR 232**

**SxtF_CR_ITEP_A3 TQLILNTAADYVLIFGKFGLPQLGLAGIGWATALGFWVSFTLGLILLIFSLKVRDYKLFR 232**

**SxtF_Raph_D9 TQLILNTAADYVLIFGKFGLPQLGLAGIGWATALGFWVSFTLGLILLIFSLKVRDYKLFR 232**

NorM_Nostoc_sp_PmC VSLLLNIPVNYVLMFGFLGFPALGLAGISWGTMLVYWVSFLASVMLIYFHPNSRDYKLFR 233

NorM_VC_RC385 IGLLLNIPLNWIFVYGKFGAPELGGVGCGVATAIVYWIMLLLLLFYIVTSKRLAHVKVFE 228

:** . ::::::* :* * ** .* . .: : :*: : :. : . . ::*

SxtM_CR_ITEP_A3 YLHQFDRQTVVEIFQTGWPMGFLLGVESVVLSLTAWLTGYLGTVTLAAHEIAIQTAELAI 293

SxtM_CR_T3 YLHQFDRQTVVEIFQTGWPMGFLLGVESVVLSLTAWLTGYLGTVTLAAHEIAIQTAELAI 300

SxtM_CR_PMC0001 YLHQFDRQTVVEIFQTGWPMGFLLGVESVVLSLTAWLTGYLGTVTLAAHEIAIQTAELAI 293

SxtM_Raph_D9 YLHQFDRQTVVEIFQTGWPMGFLLGVESAVLSLSAWLAGYLGTVTLAAHEIAIQTAELAI 300

SxtM_CR_MVCC14 YLHQFDRQTVVEIFQTGWPMGFLLGVESAVLGLSAWLAGYLGTVTLAAHEIAIQTAELAI 293

SxtM3_LW CLHQFDKQTFVKIFQTGWPMAFLLGAESVVLNVNAWLAGYLGTVTLAAHEMAMQTAAIAI 300

SxtM2_LW YWHQFDRQTFVKIFQTGWPMGFQWGAESALLSVSAGLAGYLGTVTLAAHEMAFETVEMAM 300

SxtM1_LW YWHQFDRQTFVKIFQTGWPMGFQWGAESALLSVSAGLAGYLGTVTLAAHEMAFETVEMAM 300

SxtM_AC_AWQC131C YMRQFDKQTFVKIFQTGWPMGFLLGAESVVLNVNAWLAGYVGTATLAAHEIAAQTAAMAM 300

SxtM_Apha_NH5 YMHQFDKQTFVKIFQTGWPMGIQWASETSLLNVSAWLAGYLGTVTLAAHEIAAQTAEMAM 300

**SxtF_CR_T3 YLHQFDKQIFVKIFQTGWPMGFQWGAETALFNVTAWVAGYLGTVTLAAHDIGFQTAELAM 292**

**SxtF_CR_MVCC14 YLHQFDKQIFVKIFQTGWPMGFQWGAETALFNVTAWVAGYLGTVTLAAHDIGFQTAELAM 292**

**SxtF_CR_PMC0001 YLHQFDKQIFVKIFQTGWPMGFQWGAETALFNVTAWVAGYLGTVTLAAHDIGFQTAELAM 292**

**SxtF_CR_ITEP_A3 YLHQFDKQIFVKIFQTGWPMGFQWGAETALFNVTAWVAGYLGTVTLAAHDIGFQTAELAM 292**

**SxtF_Raph_D9 YLHQFDKQIFVKIFQTGWPMGFQWGAETALFNVTAWVAGYLGTVTLAAHDIGFQTAELAI 292**

NorM_Nostoc_sp_PmC YLDEFDREIFGKIFQIGWPTGIQLAIEMGLFTITAMLMGRLGTSSLAAHEIALQASSIFS 293

NorM_VC_RC385 TFHKPQPKELIRLFRLGFPVAAALFFEVTLFAVVALLVAPLGSTVVAAHQVALNFSSLVF 288

: : : . .:*: *:* . * :: : * : . :*: :***::. : :

SxtM_CR_ITEP_A3 VIPLGIGNVAVTRVGQTIGEKNPLGARRAALIGIMIGGIYASLVAVIFWLFPYQIAGLYL 353

SxtM_CR_T3 VIPLGIGNVAVTRVGQTIGEKNPLGARRAALIGIMIGGIYASLVAVIFWLFPYQIAGLYL 360

SxtM_CR_PMC0001 VIPLGIGNVAVTRVGQTIGEKNPLGARRAALIGIMIGGIYASLVAVIFWLFPYQIAGLYL 353

SxtM_Raph_D9 VIPLGIGNVTVTRVGQTMGEKNPLGARRAALIGIMIGGIYASAVALIFWLFPYQIAGLYL 360

SxtM_CR_MVCC14 VIPLGIGNVTVTRVGQTMGEKNPLGARRAALIGIMIGGIYASAVALIFWLFPYQIAGLYL 353

SxtM3_LW VIPLGIGNVAVTRVGQTRGEKNPAGARRAALIGITFGGIYASAVALIFWLFPYQIAGIYL 360

SxtM2_LW VLPLGLGNVAVTRVGQTMGEKNPLGAKRAGLIGITIGSIYASAVAFIFWLFPYQIAGIYL 360

SxtM1_LW VIPLGIGNVAVTRVGQTMGEKNPLGAKRAGLIGITIGSIYASAVAFIFWLFPYQIAGIYL 360

SxtM_AC_AWQC131C VIPLGVGNVAVTRVAQTLGEKKPLGARRAASIGITIGAIFASAAALIFWLLPYQIAGIYL 360

SxtM_Apha_NH5 VIPLGVGNVAVTRVAQTLGEKKPLGARRAASIGITIGAIFASAAALIFWLLPYQIAGIYL 360

**SxtF_CR_T3 VIPLGVGNVAMTRVGQSIGEKNPLGARRVASIGITIVGIYASIVALVFWLFPYQIAGIYL 352**

**SxtF_CR_MVCC14 VIPLGVGNVAMTRVGQSIGEKNPLGARRVASIGITIVGIYASIVALVFWLFPYQIAGIYL 352**

**SxtF_CR_PMC0001 VIPLGVGNVAMTRVGQSIGEKNPLGARRVASIGITIVGIYASIVALVFWLFPYQIAGIYL 352**

**SxtF_CR_ITEP_A3 VIPLGVGNVAMTRVGQSIGEKNPLGARRVASIGITIVGIYASIVALVFWLFPYQIAGIYL 352**

**SxtF_Raph_D9 VIPLGVGNVAMTRVGQSIGEKNPLGARRVASIGITIVGIYASIVALVFWLFPYQIAGIYL 352**

NorM_Nostoc_sp_PmC AITMAISYAVTARVGQMMGEKNPKGVIRATFVNLSLSVLLAFVVAIGFELFSQPIATLYL 353

NorM_VC_RC385 MFPMSIGAAVSIRVGHKLGEQDTKGAAIAANVGLMTGLATACITALLTVLFREQIALLYT 348

:.:.:. .. **.: **:.. *. . :.: * .*. *: ** :*

SxtM_CR_ITEP_A3 KINDPESMEAVKTATNFLFLAGLFQFFHSVQIIVVGVLIGLQDTFIPLLMN-LVGWGLGL 412

SxtM_CR_T3 KINDPESMEAVKTATNFLFLAGLFQFFHSVQIIVVGVLIGLQDTFIPLLMN-LVGWGLGL 419

SxtM_CR_PMC0001 KINDPESMEAVKTATNFLFLAGLFQFFHSVQIIVVGVLIGLQDTFIPLLMN-LVGWGLGL 412

SxtM_Raph_D9 KINDPESMEAVKTATTFLVLAGLFQFFYSIQIIIVGALIGLQDTFMPLLMN-LVSWGLGL 419

SxtM_CR_MVCC14 KINDPESMEAVKTATTFLVLAGLFQFFYSIQIIIVGALIGLQDTFMPLLMN-LVSWGLGL 412

SxtM3_LW DINNPENIEAVKTATTFLALAGLFQFFYSIQIIIVGALIGLQDTFVPLLMN-LVGWGLGL 419

SxtM2_LW DINNPENIEAVKTATSFLILAGLFQLFYSIQVIIVGALVGLQDTLVPVLMN-LGGWGIGL 419

SxtM1_LW DINNPENIEAVKTATSFLVLAGLFQLFYSIQVITVGALIGLQDTLVPVLMN-LGGWGIGL 419

SxtM_AC_AWQC131C NINNPENIEAVKTATSFLALSGLFQIFYSIQIITVGALIGLQDTFVPLLMN-LVGWGLGL 419

SxtM_Apha_NH5 NINNPENIEAINTATSFLALSGLFQIFYSIQMITVGALIGLQDTFVPVLMN-LVVWVLGL 419

**SxtF_CR_T3 NINNPENIEAIKKATTFIPLAGLFQMFYSIQIIIVGALVGLRDTFVPVSMN-LIVWGLGL 411**

**SxtF_CR_MVCC14 NINNPENIEAIKKATTFIPLAGLFQMFYSIQIIIVGALVGLRDTFVPVSMN-LIVWGLGL 411**

**SxtF_CR_PMC0001 NINNPENIEAIKKATTFIPLAGLFQMFYSIQIIIVGALVGLRDTFVPVSMN-LIVWGLGL 411**

**SxtF_CR_ITEP_A3 NINNPENIEAIKKATTFIPLAGLFQMFYSIQIIIVGALVGLRDTFVPVSMN-LIVWGLGL 411**

**SxtF_Raph_D9 NINNPENIEAIKKATTFIPLAGLFQMFYSIQIIIVGALVGLRDTFVPVSMN-LIVWGLGL 411**

NorM_Nostoc_sp_PmC DINNPDNAVAITQAINFLKLVAVYQVFSSIQGIAVGALLGLQDTRVPMIINTLSFWGVGL 413

NorM_VC_RC385 -----ENQVVVALAMQLLLFAAIYQCMDAVQVVAAGSLRGYKDMTAIFHRTFISYWVLGL 403

:. .: * :: : .::* : ::* : .* * * :* . . : * :**

SxtM_CR_ITEP_A3 AVSYYMGII-----LCWGGMGIWLGLVLSPLLSGLILMVRFYQEIANRIANSDDGEE--- 464

SxtM_CR_T3 AVSYYMGII-----LCWGGMGIWLGLVLSPLLSGLILMVRFYQEIANRIANSDDGQESIS 474

SxtM_CR_PMC0001 AVSYYMGII-----LCWGGMGIWLGLVLSPLLSGLILMVRFYQEIANRIANSDDGEE--- 464

SxtM_Raph_D9 AVSYYMGTI-----LGWGGMGIWLGMVLSPLLSGVILMVRFYQEIAKKIANSDDGEELVL 474

SxtM_CR_MVCC14 AVSYYMGTI-----LGWGGMGIWLGMVLSPLLSGVILMVRFYQEIAKKIANSDDGEE--- 464

SxtM3_LW AGSYCMGII-----LGWGGIGIWLGMVLSPLLSGVILMVRFYQEIANKIANSDDGEQSMS 474

SxtM2_LW TGSYCMGII-----LGWGGMGIWLGMVLSPLLSGVILIARFYQAIGKKIANSDDGEESQL 474

SxtM1_LW TGSYCMGII-----LGWGGMGIWLGMVLSPLLSGVILMVRFYQAIGKKIANSDDGEESMS 474

SxtM_AC_AWQC131C AGSYFMAII-----LGWGGIGIWLGMVLSPLLSGVILMVRFYQMIAYKIANSNNGEESQI 474

SxtM_Apha_NH5 AGSYFMAII-----LGWGGIGIWLGMVLSPLLSGVILMVRFYQMIAHKIANSNNGEESQI 474

**SxtF_CR_T3 AGSYFMAII-----LGWGGIGIWLAMVLSPLLSAVILTVRFYRVIDNLLANSDDMLQNAS 466**

**SxtF_CR_MVCC14 AGSYFMAII-----LGWGGIGIWLAMVLSPLLSAVILTVRFYRVIDNLLANSDDMLQNAS 466**

**SxtF_CR_PMC0001 AGSYFMAII-----LGWGGIGIWLAMVLSPLLSAVILTVRFYRVIDNLLANSDDMLQNAS 466**

**SxtF_CR_ITEP_A3 AGSYFMAII-----LGWGGIGIWLAMVLSPLLSAVILTVRFYRVIDNLLANSDDMLQNAS 466**

**SxtF_Raph_D9 AGSYFMAII-----LGWGGIGIWLAMVLSPLLSAVILTVRFYRVIDNLLANSDDMLQNAS 466**

NorM_Nostoc_sp_PmC GGGYLMGII-----LGWGGTGLWYGLIMAPAISSLILVGRFYLRAKKFEGDLTGVPDQTP 468

NorM_VC_RC385 PIGYILGMTNWLTEQPLGAKGFWLGFIIGLSAAALMLGQRLYWLQK----QSDDVQLHLA 459

.* :. *. *:* .:::. :.::* *:* : .

SxtM_CR_ITEP_A3 -----------------------------

SxtM_CR_T3 IDNVEELS--------------------- 482

SxtM_CR_PMC0001 -----------------------------

SxtM_Raph_D9 SQFNRQTYE-------------------- 483

SxtM_CR_MVCC14 -----------------------------

SxtM3_LW NSSQLIDSSIYKRLTGLKQKISSLRLFRT 503

SxtM2_LW ILTDKVLKCRS------------------ 485

SxtM1_LW KLKHL------------------------ 479

SxtM_AC_AWQC131C IPDEKVLKCIS------------------ 485

SxtM_Apha_NH5 IPAEKVLKCIS------------------ 485

**SxtF_CR_T3 VTTLG------------------------ 471**

**SxtF_CR_MVCC14 VTTLG------------------------ 471**

**SxtF_CR_PMC0001 VTTLG------------------------ 471**

**SxtF_CR_ITEP_A3 VTTLG------------------------ 471**

**SxtF_Raph_D9 VTTLG------------------------ 471**

NorM_Nostoc_sp_PmC VSTLST----------------------- 474

NorM_VC_RC385 AK--------------------------- 461
