# Supplementary material for: In Silico Analysis of Putative Paralytic Shellfish Poisoning Toxins Export Proteins in Cyanobacteria
Source: PLoS One. 2013 Feb 15;8(2):e55664. doi: 10.1371/journal.pone.0055664 (PMC3574068; doi:10.1371/journal.pone.0055664)
Supplement: File S2 — Best amino acid substitution model identified by Akaike Information Criterion (AIC). (PDF) [file pone.0055664.s005.pdf]

```
ProtTest - 2.4                          Selection of models of protein evolution
(c) 2004                          Federico Abascal, Rafael Zardoya, David Posada
(FA, DP)             Facultad de Biologia, Universidad de Vigo, 36200 Vigo, Spain
(RZ)                    Museo Nacional de Ciencias Naturales, 28006 Madrid, Spain
Contact:                                 fedeabascal@yahoo.es, dposada@uvigo.es
--------------------------------------------------------------------------------

Wed Nov 14 19:18:06 CET 2012
OS = Linux (2.6.34.10-0.6-desktop)


ProtTest options
----------------
  Alignment file........... :
/srv/www/vhosts/darwin.uvigo.es/serving/prottest/tmp/alignment_22034
  Tree..................... : BioNJ
  StrategyMode............. : Slow (optimize branch lengths, model & topology)
     Tree search........... : NNI
  Candidate models......... :
    Matrices............... : JTT LG DCMut MtREV MtMam MtArt Dayhoff WAG RtREV
CpREV Blosum62 VT HIVb HIVw
    Distributions.......... : +I +G +I+G
    Number of rate categ... : 4
    Observed frequencies... : true
  Statistical framework
    Sort models according to....: AIC
    Sample size.................: 0.0 (not calculated yet)
      sampleSizeMode............: Total number of characters (aligment length)
  Other options:
    Display best tree in ASCII..: false
    Display best tree in Newick.: true
    Verbose.....................: true


**********************************************************
  Reading alignment...
    Sequence #1: SxtF_D9
    Sequence #2: SxtF_T3
    Sequence #3: SxtF_CRJ1
    Sequence #4: SxtF_CRA3
    Sequence #5: SxtF_CR01
    Sequence #6: SxtM1_LW
    Sequence #7: SxtM2_LW
    Sequence #8: SxtM3_LW
    Sequence #9: SxtM_D9
    Sequence #10: SxtM_T3
    Sequence #11: SxtM_AphaN
    Sequence #12: SxtM_AC_AW
    Sequence #13: SxtM_CR_J1
    Sequence #14: SxtM_CR03
    Sequence #15: SxtM_CR01
    Sequence #16: NorM_Nosto
    Sequence #17: NorM_VCRC3
   Alignment contains 17 sequences of length 449
```

****
Date   : Wed Nov 14 20:10:15 CET 2012
Runtime: 0h:52:09

****
Best model according to AIC: JTT+G+F
****

Model	deltaAIC*	AIC	AICw	-lnL
JTT+G+F	0.00	9877.95	0.53	-4887.98
CpREV+G+F	1.96	9879.91	0.20	-4888.96
JTT+I+G+F	2.00	9879.95	0.20	-4887.98
CpREV+I+G+F	3.96	9881.91	0.07	-4888.96
JTT+I+F	37.04	9914.99	0.00	-4906.50
WAG+G+F	42.88	9920.84	0.00	-4909.42
CpREV+I+F	44.88	9922.83	0.00	-4910.42
WAG+I+G+F	44.88	9922.84	0.00	-4909.42
JTT+F	47.45	9925.40	0.00	-4912.70
CpREV+F	52.71	9930.67	0.00	-4915.33
LG+G+F	66.45	9944.41	0.00	-4921.20
HIVb+G+F	68.03	9945.98	0.00	-4921.99
LG+I+G+F	68.45	9946.41	0.00	-4921.20
HIVb+I+G+F	70.03	9947.98	0.00	-4921.99
WAG+I+F	74.83	9952.78	0.00	-4925.39
WAG+F	82.83	9960.79	0.00	-4930.39
MtREV+G+F	86.64	9964.59	0.00	-4931.30
MtREV+I+G+F	88.64	9966.59	0.00	-4931.29
Dayhoff+G+F	89.63	9967.59	0.00	-4932.79
DCMut+G+F	90.20	9968.16	0.00	-4933.08
